# Subacute sclerosing panencephalitis in a child six years after measles infection

**DOI:** 10.1016/j.idcr.2026.e02528

**Published:** 2026-02-19

**Authors:** Laura Le Feur, Steven Henry, Eric Jeziorski, Julia Dina, Julie Piarroux, Pierre Meyer, Vincent Foulongne

**Affiliations:** a Virology Laboratory, Montpellier University Hospital, France; b Pathogenesis and Control of Chronic and Emerging Infections (PCCEI), University of Montpellier, Établissement français du sang, INSERM, Université des Antilles, Montpellier, France; c Department of General Pediatrics, Infectious Diseases and Clinical Immunology, Montpellier University Hospital, France; d French Measles National Reference Center, France; e Department of Pediatric Neurology, Montpellier University Hospital, France

**Keywords:** Measles, Subacute Sclerosing Panencephalitis, SSPE, Vaccination

## Abstract

Measles is a highly contagious viral illness that remains endemic in many parts of the world, posing significant risks of severe and long-term complications. We report the case of an 8-year-old boy hospitalized for epilepsy with myoclonic spasms and cognitive decline. Clinical and paraclinical investigations, including electroencephalography, imaging tests, and cerebrospinal fluid (CSF) analysis, suggested a neurodegenerative process. The patient had contracted measles at the age of two and had not received full vaccination. Elevated measles-specific immunoglobulins and intrathecal focus of synthesis, as confirmed by the French National Reference Center, confirm the diagnosis of subacute sclerosing panencephalitis. Despite initiation of antiviral and immunomodulatory treatments, the patient experienced a neurological deterioration, ultimately developing severe motor and cognitive impairment. This case highlights the severity of a rare but concerning pathology in the light of emerging measles epidemics and the decline in vaccination.

## Introduction

Measles is a highly contagious viral infection that can lead to serious complications, affecting the pulmonary, gastrointestinal, ophthalmological, and neurological systems. Among the neurological manifestations, subacute sclerosing panencephalitis (SSPE) is a rare but devastating disorder, characterized by progressive and irreversible damage to the central nervous system (CNS). This disorder results from the persistence of the measles virus in the CNS several years after the initial infection [1]. The disease often begins insidiously with early symptoms such as memory impairment, irritability, or insomnia. Over time, it progresses to myoclonic crisis, seizures, and progressive motor dysfunction resulting in autonomic failure. The persistence and transneuronal spread of the virus may be related to mutations in its genome, particularly in the M and F protein genes that could allow the virus to evade the immune system within the CNS [2]. Furthermore, some studies suggest that genetic predispositions may also contribute to the development of SSPE [3].

The incidence of SSPE is estimated to be close to 4–11 cases per 100,000 measles cases when primary infection occurs after the age of five. This risk increases significantly in children infected before the age of five, reaching 18 cases per 100,000 incidence [4]. Furthermore, SSPE remains more prevalent in countries with low vaccination coverage, notably in India, Pakistan, Papua New Guinea, and Turkey [3]. The disease predominantly affects children and adolescents and invariably progresses to severe neurodegeneration and death in the absence of curative treatment. Thanks to widespread vaccination in France and across Europe, this complication had nearly disappeared. Only a few dozen cases have been reported in the medical literature in recent years [5], [6], [7], [8]. No consolidated global figure is available for the total number of reported SSPE cases. Furthermore, SSPE is not a notifiable disease in many countries, including France, which further limits the availability of reliable epidemiological data. In 2023, only one acute encephalitis was reported within the European Union, according to the European Centre for Disease Prevention and Control (ECDC) [9]. However, the recent resurgence of measles following the advent of COVID-19 pandemic, linked to a decline in vaccination coverage in certain regions, has brought renewed concern about this serious complication.

We report a clinical case of subacute sclerosing panencephalitis that occurred in a child several years after a measles virus infection, highlighting the severe consequences that can result from inadequate vaccine protection.

## Clinical case

An 8-year-old boy was brought to the emergency department for evaluation of brief episodes of impaired awareness and spasms involving the lower limbs. These events occurred without any recent trauma, fever, vomiting, or diarrhea episodes, and with a preserved general condition. He was subsequently admitted to the pediatric neurology department for suspected epilepsy associated with focal seizures. During the initial medical history, no significant personal medical background was identified, and the family reported no consanguinity either. However, there was a family history of a single febrile seizure in the father at the age of 18, and a case of generalized epilepsy in a distant cousin.

**Fig. 1:**
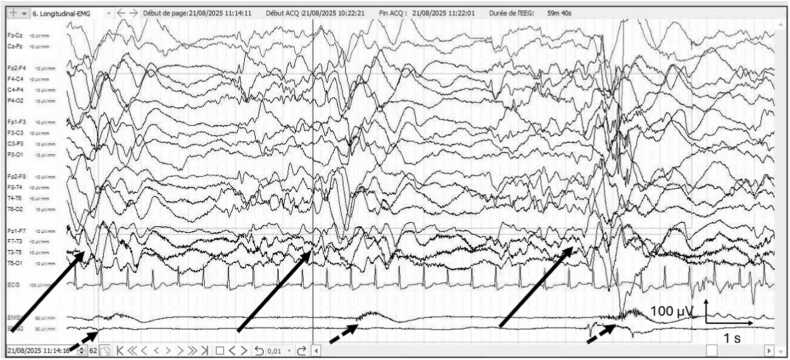
Patient’s EEG with typical periodic slow complexes (long arrows) and muscular activation under the EMG electrods, correlated with the complexes (short dotted arrows).

**Fig. 2:**
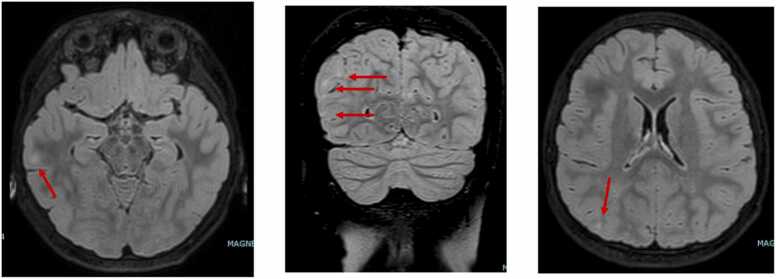
The brain MRI shows cortical and subcortical hypersignals in the right central, right temporal, and right occipital regions on the FLAIR sequence (arrows).

Upon admission, the patient was experiencing myoclonic status epilepticus and/or brief spasms despite triple antiepileptic therapy with vigabatrin, levetiracetam, and topiramate. Clinical examination revealed loss of muscle tone and disorientation in time and space. An awake electroencephalogram (EEG) (Fig. 1). Showed a poorly organized background for the patient's age, with diffuse fast rhythms and slow waves in the right posterior region. Numerous myoclonic spasms were recorded on video and electromyography (EMG), sometimes preceding a burst of abnormalities on the EEG. Brain magnetic resonance imaging (MRI) (Fig. 2) revealed FLAIR hyperintensities in the cortico-subcortical regions of the right central area, right trigone, and right temporal and occipital lobes. Additional cortical hyperintensities were suspected in the frontal convexities and anterior temporal lobes bilaterally.

An intravenous loading dose of clonazepam at 0.015 mg/kg was administered, followed by maintenance therapy via continuous perfusion, then oral administration. Simultaneously, intravenous methylprednisolone pulses were given at 20 mg/kg for 3 days, and lacosamide was introduced, resulting in a reduction of seizure frequency.

Given the neurological presentation, EEG findings (Fig. 1), and MRI abnormalities (Fig. 2), the case was reviewed during a national epilepsy multidisciplinary team meeting. The differential diagnoses considered included autoimmune disease, metabolic disorder, and SSPE secondary to measles infection.

Plasma amino acid chromatography did not reveal evidence of a metabolic disorder. Testing for anti-neutrophil cytoplasmic antibody (ANCA), onconeural, anti-NMDA, anti-MOG, and anti-ganglioside antibodies were negative, providing no support for a diagnosis of autoimmune neurological disease. The patient’s serologies for CMV, EBV, VZV, and HSV were consistent with past infections. However, in the evaluation for an inflammatory condition, IgG and albumin levels were measured in both serum and cerebrospinal fluid (CSF), resulting in a total Link index of 2.65 (reference range < 0.7). A Link index (with a normal albumin quotient) greater than 0.7 is considered indicative of intrathecal immunoglobulin synthesis [10], which was the case in our patient. This synthesis suggests a state of systemic hyperimmunization that may be associated with multiple sclerosis, a chronic inflammatory, infectious, post-infectious, or neoplastic disease of the central nervous system.

Further discussion with the parents revealed that the patient had contracted measles at the age of two, despite being unvaccinated or having received at most a single dose of the vaccine. The mention of the infectious episode was indeed found in his medical records, but no evidence of a vaccine dose being administered was found. A lumbar puncture was performed, showing a CSF cell count of 6 cells/mm³ , and PCR testing for viral pathogens: CMV (BeGenius CMV ELITe MGB, ELITech), HSV, VZV (RealStar alpha Herpesvirus, Altona), and Enterovirus (Bosphore ADEP Panel Kit, Anatolia Geneworks) was negative. Automated chemiluminescence immunoassay was carried out with Liaison XL (Diasorin) to measure IgG antibodies directed against the measles virus in both CSF and serum. Serum and CSF samples IgG dosages were highly positive with furthermore a link index > 3, suggestive for an intrathecal focalization of immunoglobulin secretion. The samples were subsequently sent to the French National Reference Center for Measles for confirmation. The results, showing a CSF/serum relative quotient (CSQrel) of 17.010 (>1.5) according to the Euroimmun test instruction (EUROIMMUN-Software-EUROLabCSF indd), indicate intrathecal synthesis of measles-specific immunoglobulins, consistent with a diagnosis of subacute sclerosing panencephalitis. The patient did not receive any immunoglobulin administration before the measurements.

Following consultation with infectious disease and immunology specialists, as well as a national multidisciplinary case review, a treatment was initiated, consisting of lamivudine 10 mg/kg/day, isoprinosine 50 mg/kg/day, and Beresmi (ropeinterferon alfa-2b) 210 μg every two weeks. A ketogenic diet was also implemented as an adjunctive therapy to limit neuronal hyperstimulation. Despite these interventions, the child's condition continued to deteriorate over the following weeks, requiring the placement of a nasogastric feeding tube and an implantable port-a-cath. Lamivudine and Beresmi were discontinued due to multiple adverse effects. An anticonvulsant treatment including valproic acid, clonazepam, and piracetam was initiated, allowing for better tolerance of the frequent daily myoclonic seizures that persisted.

The patient was subsequently transferred to a pediatric rehabilitation institute. To date, the patient has developed severe disabilities with mixed spastic quadriparesis requiring botulinum toxin injections. The other clinical conditions also included dystonic-dyskinetic movements, daily seizures, loss of language and head control, and both urinary and fecal incontinence. He also presents with severe intellectual disability, posterior sialorrhea, and need for assistance with all activities of daily living.

## Discussion

The incidence of SSPE remains difficult to estimate, as it is not a notifiable disease and is not included in global estimates of acute measles-related mortality, which is defined as death occurring within 30 days of rash onset [11]. Moreover, SSPE is likely to be underdiagnosed in developing countries due to its late onset and non-specific clinical presentation. Indeed, there is no single pathognomonic finding for the diagnosis of subacute sclerosing panencephalitis. Brain MRI may demonstrate white matter abnormalities, often with posterior predominance, however these findings are not specific. The diagnosis relies on a combination of findings. It can be established using the Dyken’s criteria, requiring evidence of intrathecal measles antibody synthesis, typical or atypical clinical history, and at least one minor criteria among typical EEG findings, characteristic histopathological findings on brain biopsy, elevated globulin levels in the CSF or evidence of mutated measles virus genome [12].

This clinical case occurs in the context of a significant resurgence of measles. According to the WHO, 127,350 cases were reported in Europe in 2024, twice the number reported in 2023 and the highest since 1997 [13]. The COVID-19 pandemic initially led to a significant decrease in measles cases [14], primarily due to the implementation of barrier measures and lockdowns. However, this period was also marked by a decline in vaccination coverage, linked not only to lockdown related disruptions but also to a rise in vaccine hesitancy fueled by misinformation about vaccines [15]. Consequently, the years 2023–2024 saw a marked resurgence in measles cases [13], thereby increasing the risk of severe complications such as SSPE. This patient’s case is not linked to the current outbreak as SSPE develops several years after measles virus infection. However, measles primary infection occurred in 2018 during a previous epidemic event in France. The 2018 epidemic resulted in numerous cases: 2919 cases were reported in France, with an outbreak in the Occitanie region [16].

No standardized recommendations define a reference treatment for SSPE. Therapeutic strategies are based on small case series and observational studies, with variable and often limited efficacy. Combination therapy, most commonly based on inosine pranobex (Isoprinosine), interferon alpha and antiviral agents, is the approach most frequently reported in the literature [6], [17]. The efficacy of these immune modulator drugs and antiviral molecules such as ribavirin or lamivudine has not been demonstrated. Still, these therapies are used to stabilize the progression of the disease [2], [18]. Measles vaccination remains the only effective strategy to prevent SSPE as it prevents the initial measles virus infection. To be fully effective, anti-measles vaccination requires a vaccination coverage rate above 95 %, a threshold that is still far from being reached, particularly in developing countries. Vaccination programs, such as the WHO’s “Immunization Agenda 2030” [19], are therefore of paramount importance in the global fight against measles-related morbidity and mortality.

## CRediT authorship contribution statement

**Laura Le Feur:** Writing – original draft, Investigation. **Steven Henry:** Writing – review & editing. **Julie Piarroux:** Writing – review & editing. **Eric Jeziorski:** Writing – review & editing. **Julia Dina:** Writing – review & editing. **Pierre Meyer:** Writing – review & editing. **Vincent Foulongne:** Writing – review & editing, Supervision.

## Declaration of Competing Interest

The authors declare that they have no known competing financial interests or personal relationships that could have appeared to influence the work reported in this paper.
